# Impact of the 2019 Novel Coronavirus Pandemic on Canadian Pediatricians

**DOI:** 10.1002/pdi3.70054

**Published:** 2026-09-25

**Authors:** Shaneela Shahid, Peter Rosenbaum, Sebastian Gheorghiu, Ivan D. Florez

**Affiliations:** ^1^ Department of Pediatrics McMaster University Hamilton Ontario Canada; ^2^ No Affiliation Canada; ^3^ Department of Pediatrics University of Antioquia Medellin Colombia; ^4^ School of Rehabilitation Science McMaster University Hamilton Ontario Canada; ^5^ Pediatric Intensive Care Unit Clinica Las Americas—AUNA Medellin Colombia

**Keywords:** burnout, COVID‐19, pandemic, pediatrician

## Abstract

The COVID‐19 pandemic has significantly impacted the well‐being and practices of healthcare workers. The primary goal of this study was to assess the rate of burnout among Canadian pediatricians during the pandemic using all three components of the Maslach Burnout Inventory—Human Services Survey scale. Three hundred and seventy‐seven pediatricians completed the survey; approximately two‐thirds of the participants were female, and the mean age was 49 years (standard deviation: 10.7). One hundred and fifty participants (39.7%) felt burned out before the pandemic. Two hundred and sixteen participants (57.1%) felt burned out during the pandemic. More than two‐thirds of pediatricians (70%) continued working during the pandemic. Less than 5% of participants were infected with COVID‐19. Multivariable logistic regression showed that when adjusted for gender and years of practice, being a younger pediatrician, working as a front‐line physician, having a perception of medium to very high level of exposure, and seeing ≥ 20 suspected cases of COVID‐19 were associated with high levels of burnout.

## Graphical abstract

The rate of burnout was increased among younger pediatricians with less than 20 years of clinical practice, those who had a high level of perception of COVID‐19 exposure, and those working as front‐line physicians, especially if they had seen 20 or more cases of suspected COVID‐19 infection.

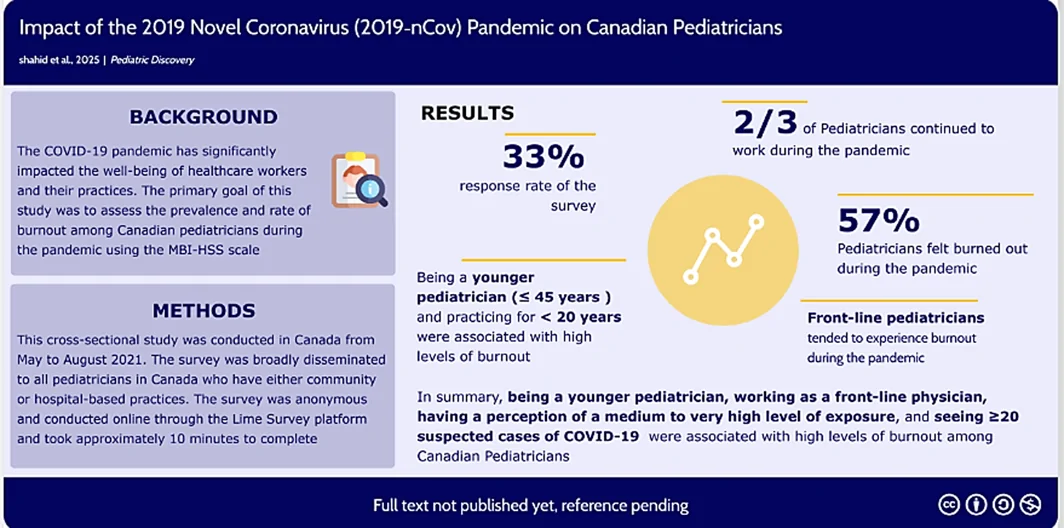

## 1 Background

Coronavirus disease (COVID‐19) is caused by the Severe Acute Respiratory Syndrome Coronavirus‐2 (SARS‐CoV‐2) [1]. In March 2020, after the WHO declared the COVID‐19 pandemic, many nations, including Canada, introduced lockdown restrictions. At the start of the pandemic, uncertainty about its seriousness created confusion, while the shortage and proper use of personal protective equipment (PPE) were also noted [2]. Many patients' services were affected, with non‐emergency surgeries and outpatient services bearing the brunt of the impact [3]. Telemedicine has rapidly emerged as a major component of clinical care, bringing both benefits and challenges [4, 5]. However, it served as a welcome option in the face of delayed care. These factors have compounded the stress experienced by healthcare providers and impacted their well‐being.

The COVID‐19 pandemic substantially affected the physical health of individuals infected with the virus and caused a psychological crisis [6, 7]. Front‐line health care providers (HCPs) caring for suspected or confirmed cases of COVID‐19 were more at risk for infection with SARS‐CoV‐2 [8]. Factors such as increased workload, caring for sick patients, limited resources, and concern about becoming infected or spreading to their family, friends, and colleagues had negative psychological impacts [8, 9, 10, 11]. Physician burnout is characterized by emotional exhaustion (EE), depersonalization (DP), and reduced personal accomplishment (PA) and has been an ongoing concern in healthcare, and the COVID‐19 pandemic has exacerbated this concern. Burnout has a direct negative impact on fatigue, anxiety, mood disorders, substance abuse, suicide, poor patient quality care, early retirement, and unexpected resignation [12].

The Canadian Medical Association (CMA) conducted a national survey in 2017, reporting that approximately 30% of physicians experienced high levels of burnout and showed symptoms of burnout, particularly EE and DP, at least weekly [13]. The CMA conducted another national health survey in 2022, reporting that over 50% of physicians experienced high burnout during the pandemic [14]. Another study among Ontario physicians using a single‐item, self‐defined burnout measure at two time periods showed that the rate of burnout increased from 28% in 2020 to 34.7% in 2021, suggesting the impact of COVID‐19 on physicians’ well‐being [15].

Among pediatricians, physician burnout has been less extensively studied. In Canada, Lee et al. conducted a mixed‐method longitudinal study from February to October 2021 among Canadian pediatric emergency physicians/fellows, which demonstrated that the predicted probability of EE was bimodal with peaks in May and October 2021, and DP rates remained low during that time [16]. This study has provided valuable insight into physician burnout in pediatrics; however, it did not include general or community pediatricians or subspecialists and used partial measures of burnout rather than a comprehensive instrument. During the pandemic, new stressors specific to pediatric practice, such as a shift from in‐person to virtual visits, increased wait times, staffing changes, and financial pressures. Moreover, limitations in PPE availability [17] and evolving clinical protocols [18] were additional stressors that may have further exacerbated the risk of burnout and its negative impact on pediatricians’ well‐being. This emphasizes the importance of examining pediatrician burnout in a comprehensive and multisetting context.

Although the literature confirms that pediatricians are at risk of burnout, there is a gap in large‐scale nationwide studies that include both hospital‐ and community‐based pediatricians, use comprehensive burnout assessment tools, and assess the impact of the COVID‐19 pandemic on pediatricians' practice. The primary objective of our study was to evaluate burnout among all practicing pediatricians in Canada by using a validated tool during the pandemic. The secondary objective was to assess the impact of the COVID‐19 pandemic on their clinical practices.

## 2 Material and Methods

This cross‐sectional study was conducted in Canada from May to August 2021. Various strategies were employed to generate the survey questions. These included reviewing the literature on burnout among pediatricians and how it was assessed before the pandemic, examining other burnout studies in other specialties during the pandemic, and adapting relevant items from them. Practice‐related questions were developed based on discussions with pediatricians and insights from published literature on the pandemic's impact on other specialties. The survey content was reviewed by three pediatric experts representing diverse practice settings (inpatient, outpatient, and acute care). To ensure clarity, content, relevance, timing, and technical issues, the survey was piloted on five pediatricians, and their feedback was incorporated into the final version. Subsequently, a final approved version of the survey was broadly disseminated to all pediatricians in Canada who have either community or hospital‐based practices or both. The survey was anonymous and conducted online through the Lime Survey platform (www.limesurvey.org) and took approximately 10 minutes to complete. Pediatricians were invited to participate in this study through various means of communication, such as email (across Canada), social media platforms such as Facebook and X (formerly Twitter), and fax/advertisement in the newsletter of a professional organization (Pediatricians of Ontario) sent to Ontario pediatricians.

Our primary outcome was the prevalence and rate of burnout in pediatricians during the pandemic, which was assessed using the Maslach Burnout Inventory—Human Services Survey (MBI‐HSS). The MBI‐HSS is a 22‐item validated tool that measures three components of burnout: EE (scores range from 0 to 54), DP (scores range from 0 to 30), and PA (scores range from 0 to 48) [19]. The EE questions screen for feeling emotionally overextended and exhausted by the physician's work. Depersonalization questions measure the presence of unfeeling and impersonal responses toward patients. Personal accomplishment questions measure feelings of competence and achievement in the physician's work. After capturing the baseline characteristics of the participants, the single‐item measure from the EE component of the MBI‐HHS scale was introduced to assess the prevalence and severity of burnout in pediatricians during the pre‐pandemic period. A single item can serve as a useful surrogate for the MBI in settings where it is not possible to administer the full 22‐item instrument [20, 21]. To assess pediatricians' burnout during the pandemic, the 22 items of the MBI‐HHS were introduced in the latter part of the survey. The MBI‐HSS is designed to measure the three levels of burnout (low, medium, and high). A high level of burnout is defined as scores in the upper third of the normative distribution for EE and DP and the lower third for PA [19]. For this study, we defined burnout as an EE score ≥ 27 and/or a DP score ≥ 10 and/or a PA of ≤ 33, which is the most frequent cutoff approach used to define burnout among physicians according to a systematic review by Rotenstein et al. [22]. Secondary outcomes included the impact of the pandemic on practice, work hours, the volume of patients, the number of hours of sleep, and the rate of influenza and COVID‐19 vaccinations among pediatricians.

In the pre‐pandemic era, the prevalence of burnout among pediatricians has been reported to be between 30% and 50%, depending on whether the practice was a community‐based pediatric practice or a pediatric critical care practice, with higher rates reported among pediatric critical care physicians [23, 24, 25]. Based on the survey published in January 2020, before the COVID‐19 pandemic, 41% of pediatricians reported burnout [26, 27]. Since we did not know the prevalence of burnout in pediatricians during the COVID‐19 pandemic, the pre‐pandemic prevalence of 40% was used for sample size calculation with a confidence interval of 95%, precision of 5%, and population of 3700 (an approximate number of Canadian Pediatric Society members in 2023) [28, 29]. Since the usual response rate of surveys is around 30%–40%, we invited over 1500 pediatricians to obtain a calculated sample size of 335 responses. Front‐line physicians in our study primarily included those who were working in the intensive care unit (ICU) or emergency room (ER), infectious disease specialists, and pediatricians working in inpatient and outpatient hospital settings, all of whom had direct patient contact during the pandemic.

Descriptive statistics were used to present pediatricians' characteristics, including age, gender, years of practice, practice location (community vs. non‐community), perception of the current level of exposure to COVID‐19, and contact with the number of suspected and confirmed cases of COVID‐19. Non‐community practice was defined as either working in an inpatient setting or seeing patients in a hospital‐based outpatient clinic. Other variables included the frequency of quarantine, the impact of the pandemic on pediatricians' practices, the number of hours of sleep before and during the pandemic, the number of working hours before and during the pandemic, and the rate of vaccination among pediatricians.

A bivariate analysis was performed to explore variables (i.e., characteristics of the pediatricians) that may have been associated with burnout. Quantitative variables are presented as the means and standard deviations (SDs) or medians and interquartile ranges (IQRs) according to their distribution, while qualitative variables are presented as frequencies and proportions. Chi‐squared tests were performed for categorical variables, and *t* tests were performed for continuous variables. A *p* value of < 0.05 was considered to indicate statistical significance.

A multivariable logistic regression model explored factors predicting the level of burnout among pediatricians. These were introduced sequentially into the model to predict the primary outcome. These predictors/factors were chosen based on evidence from similar studies on their clinical importance [25, 30, 31, 32, 33]. Data analysis was performed using IBM SPSS version 27 and Microsoft Excel 2019 edition. The Hamilton Integrated Research Ethics Board approved this study in March 2021 (ID # 13035).

## 3 Results

A total of over 1500 pediatricians were invited, of whom 1143 agreed to participate, and 377 completed the survey and were included (response rate, 33%). Approximately two‐thirds of the participants were female, and the mean age was 49 years (SD: 10.7). Over 60% (62%, 95% CI, 57%–67%) had an academic appointment with a university. About one‐third practiced in the community only, and about two‐thirds of these pediatricians (65%, 95% CI, 60%–70%) reported that they had provided coverage to hospitals. Less than 10% of participants were working in acute care settings such as ICUs and ERs. About 75% (95% CI, 70%–79%) of the respondents were front‐line clinicians. About one‐third had partners working in healthcare settings (Table 1).

**TABLE 1:** Participants' characteristics.

Characteristics	*N* (%)
Gender of pediatricians	
Female	259 (68.5%)
Male	118 (31.2%)
Age of pediatrician (mean ± SD)	49.6 (10.7) years
Year of practice (mean ± SD)	17.3 (11.3) years
Work location	
Academic	235 (62.3%)
Non‐academic	142 (37.7%)
Practice location	
Community	131 (35%)
Non‐community/hospital	246 (65%)
Working in ICU/ER	
Yes	32 (8.5%)
No	345 (91.5%)
Work position	
Front line	283 (75%)
Second line	68 (18%)
Other	26 (7%)
Partner in health care	
Yes	110 (29%)
No	267 (71%)
Perception of the current level of exposure to COVID‐19	
Very high	31 (8%)
High	64 (17%)
Medium	256 (68%)
Low	26 (7%)
Approx. # of suspected cases seen during the pandemic (median (25%–75%))	10 (3–30)
Approx. # of confirmed cases seen during the pandemic (mean ± SD)	8.14 (27.7)
Quarantine during the pandemic	
Yes	86 (23%)
No	291 (77%)
# of times quarantined	
None	291 (77%)
1–2 times	71 (19%)
3 or more	15 (4%)
# of times tested for COVID‐19	
None	121 (32%)
1–2 times	147 (39%)
3 or more	109 (29%)
COVID‐19 infection	
Yes	13 (3.4%)
No	360 (95.2%)
Influenza vaccine—2021	
Yes	358 (95%)
No	17 (4.5%)
I do not know	2 (0.5%)
COVID‐19 vaccination—2021	
Two doses	247 (65%)
At least one dose	123 (33%)
No	7 (2%)

*Note:* Approximately two‐thirds of the participants were female, and the mean age was 49.6 ± 10.7 years. Almost 75% of the respondents were front‐line clinicians. About one‐third of participants practiced in the community, and about two‐thirds of these pediatricians reported that they had provided coverage to non‐community/hospitals.

Abbreviations: ER, emergecy room; ICU, intensive care unit; SD, standard deviation.

About 25% (95% CI, 21%–29%) of participants reported that their perception of the current level of exposure to COVID‐19 (during the pandemic) was high to very high. The mean number of suspected cases of COVID‐19 and confirmed cases of COVID‐19 seen by the pediatrician was 10 (IQR 3–30) and 8 (SD: 27.7), respectively. More than 70% (77%, 95% CI, 72%–81%) of the participants did not have to quarantine during the pandemic, and for those who were quarantined, most were quarantined only 1–2 times. More than two‐thirds of pediatricians (67.7%, 95% CI, 63%–72%) were tested for COVID‐19 during the pandemic at least once, but less than 5% were found to be infected with COVID‐19. Three hundred and fifty‐eight participants (94.7%, 95% CI, 92%–97%) were vaccinated for the influenza vaccine. Two hundred and forty‐seven (65.5%, 95% CI, 60%–70%) pediatricians had received 2 doses of COVID‐19 vaccines, and 32.5% (95% CI, 27%–37%) had received at least one dose.

During the pandemic, pediatricians worked more hours per week in clinical settings (42.7 h vs. 40.6 h), with a mean increment of 2.1 h (95% CI, 1.2 to 3.0; *p* < 0.001) compared with the pre‐pandemic period. Moreover, during the pandemic, there was a reduction in the number of hours of sleep among pediatricians (6.8 h vs. 7.2 h), with a mean reduction of 0.4 h (95% CI, 0.3 to 0.5; *p* < 0.001).

### 3.1 Burnout

Before the pandemic, one hundred and fifty‐five participants (41.0%, 95% CI, 36%–46%) reported “Never” or “A few times a year or less burnout” on the single‐item measure from the EE component of the MBI‐HHS scale, indicating a low average rate of burnout [20]. One hundred and fifty participants (39.7%, 95% CI, 35%–45%) reported “Once a month or less” or “A few times a month”. Figure 1 shows the significant increase in the rate of burnout during the pandemic. During the pandemic, 42% (*n* = 159, 95% CI, 37%–47%) of participants reported “once a week” or more often to the single item measure of the EE component of the MBI‐HHS, which is considered a high average burnout rate [20], whereas only 19% (*n* = 72, 95% CI, 15%–23%) reported “Once a week” or more often to the single item measure before the pandemic. Over 55% (*n* = 216, 95% CI, 52%–62%) reported that they felt burnout during the pandemic, and 48% (95% CI, 43%–53%) of the participants had a mean score of ≥ 27 in the EE component of the MBI‐HHS, indicating a high rate of burnout (Table 2).

**FIGURE 1:**
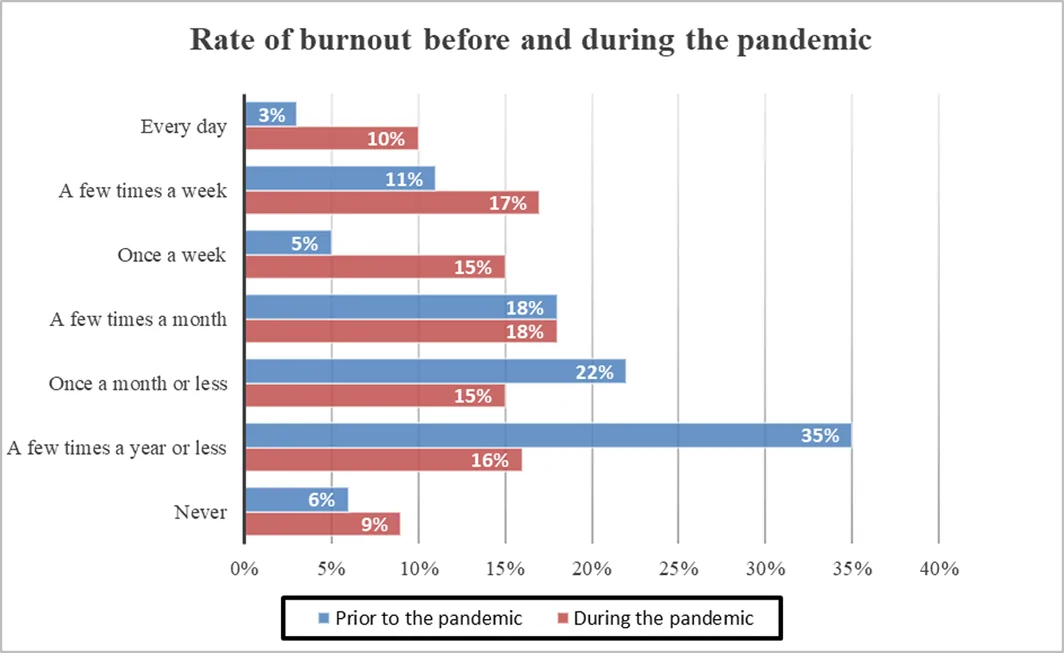
Comparison of burnout rates before and during the pandemic among pediatricians. The rate of burnout increased substantially among pediatricians during the pandemic compared to the pre‐pandemic period.

**TABLE 2:** Burnout among pediatricians during the pandemic using the MBI‐HHS tool.

MBI‐HHS	*n* (%)
Emotional exhaustion (score of ≥ 27)	183 (48)
Depersonalization (score of ≥ 10)	98 (26)
Personal accomplishment (score of ≤ 33)	82 (22)
Burnout (EE ≥ 27 and/or DP ≥ 10 and/or PA ≤ 33)	216 (57)

*Note:* Over 55% reported that they felt burnout during the pandemic, and 48% of participants had a mean score of ≥ 27 in the emotional exhaustion component of the MBI‐HHS, which is considered a high level of burnout.

Abbreviations: DP, depersonalization; EE, emotional exhaustion; MBI‐HSS, Maslach Burnout Inventory—Human Services Survey; PA, personal accomplishment.

Table 3 shows the bivariate analysis of participants' characteristics and burnout during the pandemic. Being a younger pediatrician (≤ 45 years) and practicing for less than 20 years were associated with high levels of burnout (both *p* < 0.001) during the pandemic. Pediatricians who had worked as front‐line pediatricians tended to experience burnout during the pandemic (*p* < 0.01). Moreover, seeing more than 20 suspected cases of COVID‐19 was associated with a high level of burnout (*p* < 0.01). When adjusted for the gender of the pediatrician and years of practice, multivariable logistic regression showed that being a younger pediatrician, working as a front‐line physician, having a perception of medium to very high level of exposure to COVID‐19 infection, and seeing more than 20 suspected cases of COVID‐19 were significantly associated with high levels of burnout among pediatricians during the pandemic (Table 4).

**TABLE 3:** Bivariate analysis—pediatricians' characteristics and rate of burnout in pediatricians.

Pediatricians characteristics	No burnout	Burnout	*X* ^2^ (*p* value)
	*N* (%)	*N* (%)	
Age			
Less than 45 years	48	101	
45 years or more	113	115	**11.082 (*p*** < **0.001)**
Gender			
Female	104	155	
Male	57	61	2.2 (*p* = 0.138)
Years of practice			
≤ 20 years of practice	85	151	
> 20 years of practice	76	65	**11.537 (*p* < 0.001)**
Work position			
Front line	109	174	
Second line and other	52	42	**8.143 (*p*** < **0.004)**
Work location			
Academic	103	132	
Non‐academic	58	84	0.322 (*p* = 0.570)
Work location			
Community	54	77	
Non‐community	107	139	0.181 (*p* = 0.671)
Partner in health care			
Yes	46	64	
No	115	152	0.050 (*p* = 0.823)
Perception of the level of exposure to COVID‐19			
Low to moderate	43	52	
High to very high	118	164	0.340 (*p* = 0.560)
# of suspected cases of COVID‐19 seen			
20 or less	127	143	
More than 20	34	73	**7.294 (*p*** < **0.007)**
# of confirmed cases of COVID‐19 seen			
20 or less	152	201	
More than 20	9	15	0.284 (*p* = 0.594)

*Note:* *p*‐values were calculated using chi‐square tests.

**TABLE 4:** Multivariable regression model for study variables to predict burnout in pediatricians during the pandemic.

Variables	Odds ratio	95% confidence interval	*p* value
Female gender	0.858	0.386–1.333	0.524
Age in years	0.959	0.925–0.992	**0.013**
20 or more years of practice	0.908	0.206–1.610	0.788
Work position (non‐frontline)	0.502	0.010–0.993	**0.008**
Perception of the level of exposure to COVID‐19	2.163	1.596–2.729	**0.007**
# of suspected cases of COVID‐19	2.104	1.553–2.654	**0.008**

*Note:* Bold values indicate statistically significant *p* values (*p* < 0.05).

### 3.2 Impact of the COVID‐19 Pandemic on Clinical Practice

More than two‐thirds of the pediatricians (*n* = 264, 69.8%, 95% CI, 65%–74%) reported that they continued working during the pandemic and did not have to close their practice. Only 47 pediatricians (12.4%, 95% CI, 9%–16%) had to close their practice for less than 1 month during the pandemic; and only 1% of pediatricians had to permanently close their practice. Over 300 (79.4%, 95% CI, 76%–84%) pediatricians reported that in‐person visits were reduced, and more than two‐thirds of them (75%, 95% CI, 70%–79%) reported at least a 50% reduction in the number of in‐person patient visits (Figure 2). More than 85% (95% CI, 82%–90%) of pediatricians reported an increase in virtual patient visits, and 57% (95% CI, 51%–61%) of them reported a 50% or more increment in the number of virtual visits.

**FIGURE 2:**
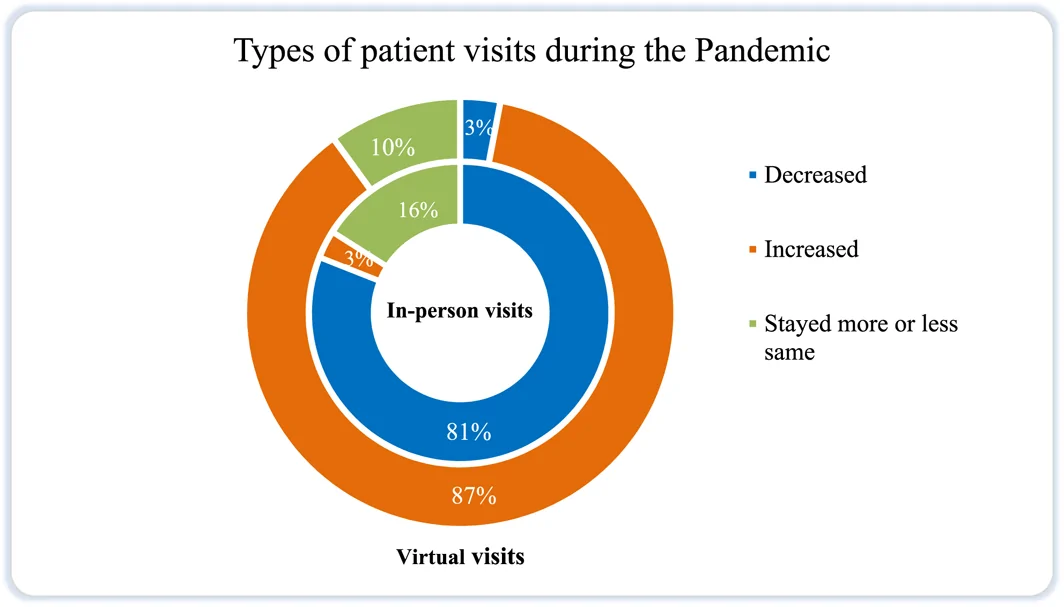
Comparison of in‐person and virtual medical visits during the pandemic. There was a reduction in the number of in‐person patient visits and an increase in virtual patient visits during the pandemic.

One hundred and forty‐seven (about 39%, 95% CI, 34%–44%) pediatricians reported no change in the wait time for seeing patients; however, 35% (95% CI, 30%–40%) reported an increase in the wait time for seeing patients during the pandemic. Over 200 pediatricians (58.9%, 95 CI, 53%–63%) reported no changes in office staff, and seventy‐two (19.0%, 95% CI, 15%–23%) reported a decrease in office staff by atleast 25%. Two hundred and eighty‐nine pediatricians (76.6%, 95 CI, 72%–80%) agreed that they felt financially stable and able to manage office expenses. Over half of the pediatricians (59%, 95% CI, 54%–64%) disagreed that they received financial support from government/professional organizations during the pandemic, whereas 108 pediatricians (about 29%, 95% CI, 24%–33%) received financial support from governmental/professional organizations, including loans (Figure 3).

**FIGURE 3:**
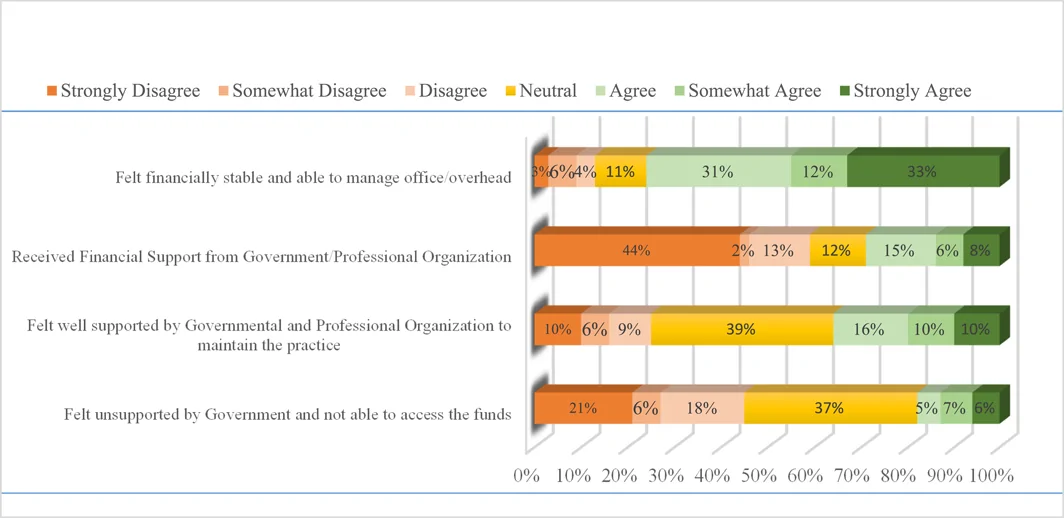
Level of agreement using the Likert Scale. More than two‐thirds of pediatricians agreed that they felt financially stable and able to manage office expenses, and about one‐third of pediatricians agreed that they received financial support from governmental/professional organizations, including loans.

## 4 Discussion

This study highlights the importance of the impact that the COVID‐19 pandemic had on Canadian pediatricians' well‐being. To the best of our knowledge, this is the first cross‐sectional study to report on the impact of the COVID‐19 pandemic on Canadian pediatricians, including the rate of burnout. The high average burnout rate increased twofold during the COVID‐19 pandemic among pediatricians compared with the rate before the pandemic. The rate of burnout was higher among younger pediatricians and pediatricians with less than 20 years of clinical practice/experience, those who had a high level of perceived COVID‐19 exposure, and those who worked as front‐line physicians, especially if they had seen 20 or more cases of suspected COVID‐19 infection.

Burnout is conceptualized as a result of chronic stress, and it has three dimensions, including chronic exhaustion, a negative attitude toward work, and professional inefficiency [34]. Burnout in physicians may lead to decreased well‐being and an increased risk of medical errors [35, 36, 37]. Physician burnout is well reported in the medical literature; however, concerns related to physician burnout have increased significantly during the COVID‐19 Pandemic [38, 39, 40]. The results of our study on the rate of burnout among pediatricians concur with the National Physician Burnout & Depression Report 2022, which surveyed over 13,000 physicians from 29 specialties working in the United States, showed high rates of burnout among pediatricians (49%) and reported pediatrics among the top 10 specialties with the highest rates of burn out after 3 years of COVID‐19 pandemic management [41].

The literature suggests that older pediatricians experience a lower level of burnout than younger pediatricians [42, 43]. Similarly, we found that being a pediatrician under 45 years of age was associated with a higher rate of burnout than being an older pediatrician. A likely explanation of this finding is the lack of self‐care strategies among young pediatricians during stressful times, as that has been previously reported [42]. Studies among non‐pediatric physicians have reported a higher prevalence of burnout in female physicians than in their male counterparts [44, 45]. Stamer et al. conducted a study before the pandemic among pediatricians and reported that female gender did not have an association with burnout [46]; similarly, our study shows that female gender was not associated with a higher rate of burnout during the pandemic.

Due to the increased rate of burnout during the COVID‐19 pandemic, burnout could be considered a COVID‐19 pandemic‐related health condition affecting an increasing number of primary care physicians, including pediatricians [47]. Possible explanations for the high rate of burnout among pediatricians during the pandemic are exposure to the COVID‐19 infection at work: fear and guilt of taking the infection home, access to childcare during school closure, financial instability, lack of work‐life balance, lack of established practices to deal with the pandemic, concerns of occupational hazards, uncertainty about organizational support, cognitive fatigue, and overall loss of autonomy [48, 49, 50, 51, 52]. Studies have reported that the rate of burnout was higher among those who worked in ICU and ER settings during the pandemic [53, 54, 55, 56]; however, in our study, very few participants worked in ICU/ER settings, and we were thus unable to draw any conclusions for this group. Studies have shown that sleep deprivation [57, 58, 59, 60] and increased workload [61, 62, 63] are associated with a higher prevalence of burnout among health care workers (HCWs). In this survey, Canadian pediatricians reported working longer hours and getting less sleep during the pandemic, which may have contributed to increased burnout.

Frontline HCWs were at an increased risk of burnout during the COVID‐19 pandemic, as they faced with many challenges, including COVID‐19 exposure risk, and occupational stressors, such as lack of hospital infrastructure, scarcity of oxygen supply, lack of ventilator support and personal protection equipment, long and stressful working hours, increased workload and stigmatization, with people viewing physicians as the possible source of spreading the infection [64, 65, 66]. In our survey, more than two‐thirds of physicians were front‐line HCWs, and being in a frontline position was associated with a higher rate of burnout. There are no published studies among pediatricians to support this finding; however, a survey from frontline physicians in the United States has shown that frontline HCWs experienced a higher rate of burnout, particularly those who have worked as hospitalists [67]. A systematic review conducted on the prevalence of burnout in physicians showed that frontline workers and HCWs displayed higher rates of burnout than second‐line healthcare workers [68]. This highlights the importance of the psychological well‐being of frontline HCWs during stressful events and the need for the allocation of resources and strategies to alleviate stress among frontline HCWs during a prolonged pandemic.

The impact of COVID‐19 on the clinical practice of pediatricians has been well reported [69, 70, 71], particularly the model of providing clinical care to patients (virtual vs. in‐person visits). These studies reported a reduction in the number of in‐person visits with an increase in virtual visits since public restrictions were in place to avoid the spread of infection. Interestingly, our study showed almost the same proportion of increment in virtual visits as there was a decline in in‐person visits. Moreover, our study showed that most pediatricians continued to work during the pandemic, and a small proportion had to temporarily close their practice. The findings of our survey concur with the study published by Piche‐Renaud et al. in Ontario Canada [72]; however, in the Piche‐Ranaud et al. study, most of the pediatricians who participated had practiced in Ontario and had practiced in the community setting, whereas in our study, participants were included across Canada, and only about one‐third had practiced in the community, which might have affected the results. Studies have shown the role of telemedicine in physician burnout and have found a lower prevalence of burnout among physicians who have used telemedicine during the COVID‐19 pandemic, reducing the prevalence of burnout and having the potential to promote provider well‐being [73, 74].

There are several strengths to our study. Burnout was assessed using the validated tool, allowing these results to be compared with other studies that have used similar tools. We also performed a regression analysis to explore the predictors associated with an increased rate of burnout in pediatricians. Moreover, this study also highlights the impact of the COVID‐19 pandemic on the clinical practice of pediatricians. In our study, about two‐thirds of participants were female pediatricians, consistent with the 2019 CMA survey findings, which reported that 63% of respondents were female and 40% practiced in Ontario [75]. Accordingly, the results of this study appear to be generalizable.

However, there are also some limitations. Firstly, this was a cross‐sectional study, which cannot establish causality between the independent variable and the outcomes. We made every effort to invite all practicing pediatricians in Canada to participate in this study to minimize selection bias. However, pediatricians working in rural communities, particularly outside Ontario, may not have participated in the study, as they were difficult to reach. Most of the participants in this survey had practice/work locations in Ontario. In this survey, we collected information about pediatricians' burnout before the pandemic; as such, there are concerns about recall bias in the reporting of pediatricians’ burnout in the pre‐pandemic period. Although the one‐item measure to assess EE could be used as a surrogate marker when the full MBI‐HHS scale cannot be used, authors recognize that this is one of the limitations.

In conclusion, the COVID‐19 pandemic significantly impacted pediatricians’ well‐being and clinical practice. The rate of burnout was increased among younger pediatricians and pediatricians with less than 20 years of clinical practice/experience, those who had a high level of perception of COVID‐19 exposure, and those working as front‐line physicians, especially if they had seen 20 or more cases of suspected COVID‐19 infection. During the pandemic, telemedicine has evolved and become the cornerstone of providing medical care among pediatricians. Most pediatricians were able to continue to provide consultations/medical care through virtual visits.

### 4.1 Future Direction and Research

This study highlights the rate of burnout among pediatricians in Canada before and during the COVID‐19 pandemic. Despite its limitations, the elevated burnout rate among pediatricians before the pandemic suggests that pediatricians’ well‐being is either inadequately addressed or underestimated. This could reflect a lack of accessible mental well‐being interventions at the individual/organizational level to take care of their well‐being or reluctance from pediatricians to seek or utilize mental well‐being support. Given the high rate of burnout both before and during the pandemic, there is a need for early and proactive mental health support for pediatricians to alleviate occupation‐related stress and exhaustion, thereby promoting the well‐being of pediatricians. This study highlights the importance and need to formulate evidence‐based interventions that improve the psychological well‐being of pediatricians, particularly frontline HCWs. At the individual level, approaches may include self‐care, identifying potential stressors, gratitude journaling, accessing mental health services, and incorporating mindfulness or meditation or yoga regularly. At the organization level, strategies may include reducing the workload by having a medical assistant, job crafting, having peer support networks, and integration of mental health experts within the organization [76]. Future research is needed to evaluate the efficacy of the above‐mentioned interventions, ideally through a randomized controlled trial (RCT) to improve physicians’ well‐being, including the reduction of work‐related exhaustion. As telemedicine has evolved during the COVID‐19 pandemic, future studies should also explore its role in enhancing patient outcomes and improving clinician well‐being across specialties, including pediatrics.

## Author Contributions

Shaneela Shahid participated in the study concept and design, contributed to the acquisition, linkage, analysis, and interpretation of data, drafted and reviewed the manuscript, and approved the final version. Ivan D. Florez participated in the study design, contributed to data acquisition, linkage, analysis, and interpretation, drafted and reviewed the manuscript, and approved the final version. Peter Rosenbaum contributed to the study design, data analysis, interpretation of the data, and manuscript review and approved the final version. Sebastian Gheorghiu contributed to the study design, acquisition, interpretation of the data, and manuscript review and approved the final version.

## Funding

The authors have nothing to report.

## Ethics Statement

The Hamilton Integrated Research Ethics Board approved this study in March 2021 (ID # 13035).

## Conflicts of Interest

Ivan D. Florez is a member of *Pediatric Discovery* Editorial Board. To minimize bias, he was excluded from all editorial decision‐making related to the acceptance of this article for publication. The other authors declare no conflicts of interest.

## Data Availability

The data that support the findings of this study are available on request from the corresponding author. The data are not publicly available due to privacy and ethical restrictions.
